# Predictive Biomarkers for Immune Checkpoint Inhibitors in Metastatic Breast Cancer

**DOI:** 10.1002/cam4.3550

**Published:** 2020-12-12

**Authors:** Abirami Sivapiragasam, Prashanth Ashok Kumar, Ethan S. Sokol, Lee A. Albacker, Jonathan K. Killian, Shakti H. Ramkissoon, Richard S. P. Huang, Eric A. Severson, Charlotte A. Brown, Natalie Danziger, Kimberly McGregor, Jeffrey S. Ross

**Affiliations:** ^1^ Upstate Medical University Syracuse New York USA; ^2^ Foundation Medicine Cambridge Massachusetts USA; ^3^ Foundation Medicine Morrisville North Carolina USA; ^4^ Wake Forest Comprehensive Cancer Center and Department of Pathology Wake Forest School of Medicine Winston‐Salem North Carolina USA

**Keywords:** biomarkers, comprehensive genomic profiling, immunotherapy, metastatic breast cancer, microsatellite instability, PD‐L1, tumor mutational burden

## Abstract

We examined a large dataset of female metastatic breast cancers (MBCs) profiled with comprehensive genomic profiling (CGP) to identify the prevalence and distribution of immunotherapy responsiveness‐associated biomarkers. DNA was extracted from 3831 consecutive MBCs: 1237 (ER^pos^/*HER2* ^neg^), 1953 ER^neg^/*HER2* ^amp^, and 641 triple‐negative breast cancer (TNBC). CGP was performed using the FoundationOne^®^ or FoundationOne^®^CDx NGS assay. Tumor mutational burden (TMB) and microsatellite instability (MSI) were determined in a subset of cases. PD‐L1 expression in immunocytes in a subset of cases was determined by immunohistochemistry using the companion diagnostic VENTANA PD‐L1 SP142 Assay. The median age of the cohort was 54 years (range 20–89). Genomic alterations (GAs)/tumor were similar (range: 5.9–7.3). Markers of potential immune checkpoint inhibitor (ICPI) benefit included: *CD274* (PD‐L1) amplification (1%–3%), *BRAF* GA (1%–4%), TMB of ≥10 mutations/Mb (8%–12%), MSI‐high (0.1%–0.4%), *PBRM1* GA (1%), and positive PD‐L1 staining of immunocytes ranging from 13% in ER^pos^/*HER2* ^neg^ and 33% in ER^neg^/*HER2* ^amp^ to 47% in the TNBC group. Potential markers of ICPI resistance included inactivating *STK11* GA (1%–2%) and *MDM2* amplification (3%–6%). MTOR pathway targets were common with lowest frequency in TNBC. *ERBB2* short variant mutations were most frequent ER^pos^/*HER2* ^neg^ and absent in TNBC. *BRCA1*/*2* GA were least frequent in ER^neg^/*HER2* ^amp^. The demonstrations of clinical benefit of immunotherapy in MBC support the need for development and utilization of biomarkers to guide the use of ICPIs for these patients. In addition to guiding therapy selection, CGP shows potential to identify GA linked to response and resistance to ICPI in MBC.

## Graphical abstract

Both our results and published literature suggest that in addition to guiding targeted therapy selection, comprehensive genomic profiling shows potential to identify genomic alterations linked to response and resistance to immune checkpoint inhibitor (ICPI) therapy in metastatic breast cancer (MBC). The demonstration of clinical benefit of immune checkpoint blockade in MBC supports the need for the development of biomarkers used to guide the use of ICPI drugs for these patients.

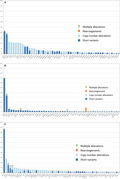

## 1 INTRODUCTION

The use of immune checkpoint inhibitors (ICPIs) targeting pathways that promote cancer immune evasion has growing momentum in the field of cancer treatment and research.^1^ Each cancer has varying degrees of somatic mutations that are unique to the cancer type, and ICPIs have been found to be more effective against tumors with high mutational burden.^2^ Exposure to carcinogens, such as smoking and UV light, has been linked to high mutation burden, and ICPIs were initially approved in cancers associated with these causes, including melanoma, non‐small cell lung cancer (NSCLC), and bladder cancer.^3^, ^4^

Over time, it was noted that ICPIs were effective only in a limited number of patients, and significant and severe immune‐related adverse effects (irAEs), such as pneumonitis, colitis, and thyroiditis, were being observed.^5^ Despite the promising clinical effects of ICPIs, the overall response rates (ORRs) have been low.^6^ For instance, pembrolizumab, which has been approved by the United States (US) Food and Drug Administration (FDA) for use in platinum refractory head and neck squamous cell carcinoma, has an ORR of only 13%–18%. It is also seen that across all cancers, resistance to ICPIs against PD‐1 and PD‐L1 approaches 60%.^7^ Recent studies have been focusing on identifying biomarkers that can help delineate patients who may have a better response to ICPIs as well as to avoid unwanted irAEs.^8^ At the same time, previous studies performed on a wide variety of malignancies suggested that PD‐L1 overexpression was a major biomarker for predicting benefit for anti‐PD‐1/PD‐L1 therapies.^9^, ^10^ The US FDA has approved the use of pembrolizumab in solid tumors with high microsatellite instability (MSI), based on a biomarker assessment of MSI status. High MSI is associated with increased number of mutations in tumoral DNA which correspond to higher levels of tumor mutational burden (TMB) and the increased presence of circulating antitumor lymphocytes and neoantigens.^11^ Neoantigens are defined as tumor‐specific antigens that arise from non‐synonymous mutations and other genetic alterations,^12^ and when processed into short peptides and presented by MHC molecules to T cells stimulate the immune system recognition of cancer cells as “non‐self” and enable subsequent immune‐mediated attack.^13^ The more somatic mutations a tumor has, the more neoantigens are likely to form, thus, TMB can be considered as an indirect measure of tumor neoantigen load. Studies have shown that tumors featuring higher TMB levels have enhanced responsiveness to ICPIs especially when they are administered as monotherapies.^14^ Based on data from the KEYNOTE‐158 trial, FDA granted regulatory approval for the use of FoundationOne CDx as the first companion diagnostic for the anti‐PD‐1 therapy pembrolizumab, to identify patients with unresectable or metastatic TMB high (≥10 mutations/megabase, mut/Mb) solid tumors that have progressed following prior treatment with no alternative treatment options.^15^

More recently, single gene mutations have been reported as predictive biomarkers for ICPI response. *BRAF* mutations have been linked to improved ICPI response, and tumors such as renal cell carcinoma with loss of function genomic alterations (GAs) in the *PBRM1* chromatin remodeling tumor suppressor gene may also be ICPI responsive.^16^, ^17^ Studies of *KRAS*‐mutant NSCLC have linked inactivating alterations in the *STK11*/*LKB1* tumor suppressor gene with significant risk of PD‐1 and PD‐L1 inhibitor resistance.^18^ *PTEN* alterations have also been associated with lower ORR and shorter progression‐free survival (PFS) independently of clinical factors and PD‐L1 status in triple‐negative breast cancer (TNBC) patients treated with ICPI.^19^ In addition, studies have indicated that the *MDM2* proto‐oncogene, a negative regulator of the *TP53* gene, when amplified as seen in multiple tumors, may be associated with disease hyperprogression in patients treated with PD‐1/PD‐L1 inhibitors.^20^

Both TNBC and HER2‐positive tumors have been recently recognized as being lymphocyte‐rich and are accompanied by abundant tumor‐infiltrating lymphocytes (TILs), also referred to as tumor‐infiltrating immune cells (ICs). PD‐L1 is expressed in the TILs of 20% of breast cancers, with TNBC and HER2‐positive tumors showing higher levels at 33% and 56%, respectively.^21^ A number of trials evaluating pembrolizumab and atezolizumab, both as monotherapy and in combination with other conventional treatment options, are in various stages and have shown notable results.^22^ In March 2019, the FDA approved atezolizumab for PD‐L1‐ positive (IC score based) unresectable locally advanced or metastatic TNBC employing the PD‐L1 assay device called VENTANA PD‐L1 (SP142) Assay as a companion diagnostic biomarker.^23^ Based on this background information, we queried whether comprehensive genomic profiling (CGP) of the three major subtypes of metastatic breast cancer (MBC) could identify biomarkers that have been linked to responsiveness to ICPI treatment.

## 2 METHODS

Approval for this study, including a waiver of informed consent and a HIPAA waiver of authorization, was obtained from the Western Institutional Review Board (Protocol No. 20152817). DNA was extracted from formalin‐fixed, paraffin‐embedded tissue samples obtained from 3831 cases of clinically diagnosed MBC received between September 2012 and July 2018, including 1237 ER+/HER2 not amplified, 1953 ER‐/HER2 amplified (amp), and 641 TNBC cases. CGP was performed using either the FoundationOne^®^ or FoundationOne^®^CDx assay in a Clinical Laboratory Improvement Amendments (CLIA)‐certified, CAP (College of American Pathologists)‐accredited laboratory (Foundation Medicine). The pathologic diagnosis of each case was confirmed on routine hematoxylin and eosin (H&E)‐stained slides and all samples forwarded for DNA extraction contained a minimum of 20% tumor nuclear area, compared with benign nuclear area.

Sequencing for the detection of base substitutions, insertions, deletions, copy number alterations (focal amplifications and homozygous deletions), and select gene fusions was performed using a hybrid capture‐based system as previously described.^24^ TMB was determined on 0.8 to 1.1 Mb of sequenced DNA for each case based on the number of somatic base substitution or indel alterations per Mb after filtering to remove known somatic and deleterious mutations, as previously described.^25^ MSI was calculated as previously described.^26^ Patients were classified as MSI‐high (MSI‐H), MSI cannot be determined, or microsatellite stable. PD‐L1 expression was determined using the VENTANA PD‐L1 (SP142) Assay on 5‐micron tissue sections. Immunohistochemistry (IHC) slide evaluation was based on the tumor‐infiltrating IC score and did not include the tumor cell score (TC). In this study, an IC ≥1% was considered to be positive. The IC score is defined as the proportion of tumor‐infiltrating IC staining in the total tumor area. Tumor areas are defined as the TCs with intra‐ and peritumoral stroma. The Fisher's exact test was used to determine if the proportion of each metastatic sample site differed across the three MBC subgroups. A *p* < 0.05 is considered statistically significant.

## 3 RESULTS

The distribution of metastatic disease sites for the majority of samples provided for CGP among the ER^pos^/*HER2* ^neg^, ER^neg^/*HER2* ^amp^, and TNBC subgroups is summarized in Table S1. The most common metastatic sites across subgroups included liver, bone, and lung. The incidence of bone metastases was significantly higher in the ER^pos^/*HER2* ^neg^ subgroup compared with the ER^neg^/*HER2* ^amp^ (10.82% vs. 4.65%, *p* < 0.0001) and TNBC subgroup (10.82% vs. 3.27%, *p* = 0.0002). The ER^neg^/*HER2* ^amp^ subgroup had a significantly higher percentage of brain metastases compared with the ER^pos^/*HER2* ^neg^ subgroup (8.90% vs. 1.59%, *p* < 0.0001).

The clinical and CGP findings in the 3831 cases of MBC are shown in Table 1. The median ages and age ranges of the patients in each subtype of MBC were similar. The mean number of GA per tumor was also similar, ranging from 5.9 GA/tumor in the TNBC group to 7.3 GA/tumor in the HER2‐amplified/ER‐ group. The known and likely GA listed by alteration type (short variant (SV) substitutions including base substitutions, short indels and truncations, copy number changes including both amplifications and homozygous deletions and rearrangements/fusions), and the protein and transcript effects are provided for the three MBC tumor types in Table S2 (ER^pos^/*HER2* ^neg^ subset of 1214 cases), Table S3 (ER^neg^/*HER2* ^amp^ subset of 1927 cases), and Table S4 (TNBC subset of 633 cases).

**TABLE 1:** Clinical features and biomarkers associated with immunotherapy responsiveness in metastatic breast cancer

	ER^pos^/HER2^neg^	ER^neg^/HER2^amp^	TNBC
Number of cases	1237	1953	641
Age (range in years)	55 (23–89)	55 (20–89)	53 (20–85)
GA/tumor	6.3	7.3	5.9
MTOR GA	*PIK3CA 38%*	*PIK3CA 38%*	*PIK3CA 19%*
	*PTEN 10%*	*PTEN 5%*	*PTEN 15%*
	*NF1 5%*	*NF1 7%*	*NF1 8%*
*CDH1* GA	7%	4%	3%
*ESR1* GA	15%	6%	0.5%
*BRCA1*/*2* GA	3%/6%	2%/3%	7%/3%
*ERBB2* amp	9%	100%	0%
*ERBB2* SV	9%	7%	0%
Other kinase targets	*FGFR1* 18%	*FGFR1* 11%	*FGFR1* 8%
	*FGFR2* 2%	*FGFR2* 1%	*FGFR2* 4%
	*EGFR* 2%	*EGFR* 3%	*EGFR* 4%
	*KIT* 1%	*KIT* 2%	*KIT* 2%
	*MET* 0.4%	*MET* 1%	*MET* 1%
	*BRAF* 2%	*BRAF* 1%	*BRAF* 4%
*AR* amp	1%	1%	1%
MSI‐High	0.2%	0.1%	0.4%
*CD274* (*PD*‐*L1*) amp	1%	1%	3%
TMB >10 mut/Mb	8%	12%	9%
TMB >20 mut/Mb	2%	2%	3%
Positive (≥1%) Immunocyte PD‐L1 IHC staining^a^	13%	33%	47%
*PBRM1* GA	1%	1%	1%
*STK11* GA	1%	1%	2%
*MDM2* amp	6%	5%	3%

Abbreviations: Amp, amplification; ER^pos^, estrogen receptor positive; GA, genomic alteration; IHC, immunohistochemistry; SV, short variant; TMB, tumor mutational burden; TNBC, triple‐negative breast cancer.

^a^ Subset of recent MBC cases that were stained for PD‐L1 immunocyte expression with the VENTANA PD‐L1 (SP142) Assay approved in March 2019 as a CDx for the atezolizumab‐nab‐paclitaxel approval for the treatment of TNBC.

**FIGURE 1:**
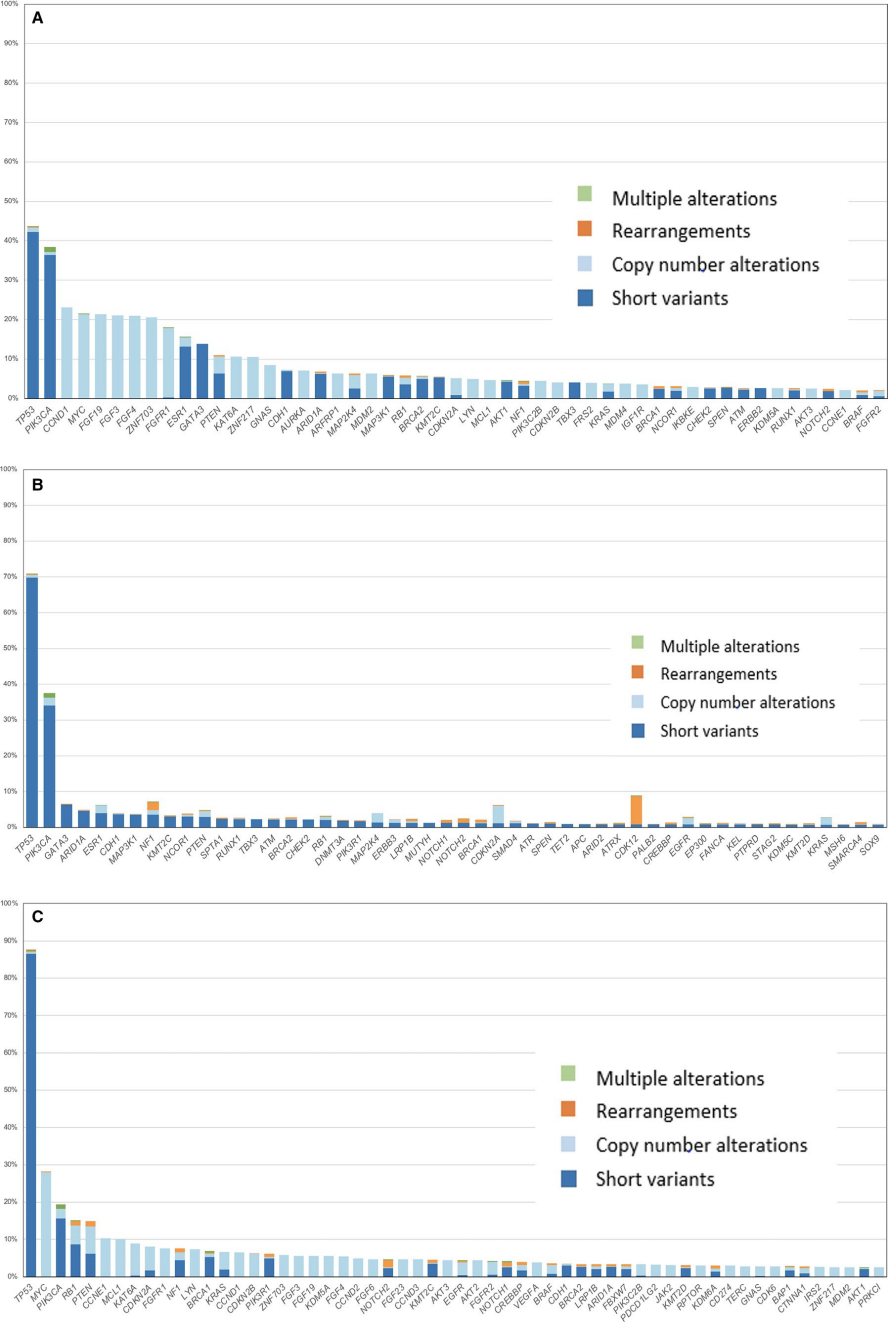
Most prevalent genomic alterations by gene and alteration type across breast cancer subtypes. (A) Genomic alterations in 1237 ER^pos^/*HER2* ^neg^ metastatic breast cancers. (B). Genomic alterations in 1953 ER^neg^/*HER2* ^amp^ metastatic breast cancers. (C) Genomic alterations in 641 triple‐negative breast cancers

As seen in Table 1 and the long tail plots (Figure 1A‐C), MTOR pathway genes (*PIK3CA*, *PTEN*, and *NF1*) were found altered in all three MBC groups, but representation was lowest in the TNBC patients. *PTEN* alterations, including SVs, deletions, and rearrangements, were detected across all three subtypes (5%–15%). *CDH1* (7%) and *ESR1* (15%) GA were most frequently identified in the ER^pos^/*HER2* ^neg^ cases. *ERBB2* SV mutations were most frequently found in the ER^pos^/*HER2* ^neg^ (9%) and in the ER^neg^/*HER2* ^amp^ cases (7%) and were not identified in TNBC cases. Other kinase targets were uncommon in all three groups except for the identification of *FGFR1* GA in ER^pos^/*HER2* ^neg^ tumors (18%). Potentially targetable kinase GA rarely encountered in any of the MBC groups included *FGFR2* (1%–4%), *EGFR* (2%–4%), *KIT* (1%–2%), *MET* (0.4%–1%), and *BRAF* (1%–4%). At 7%, *BRCA1* GA were most frequent in the TNBC cases and were rare in the ER^pos^/*HER2* ^neg^ (3%) and ER^neg^/*HER2* ^amp^ (2%) groups. At 6%, *BRCA2* GA were most frequent in the ER^pos^/*HER2* ^neg^ cases and less common in the ER^neg^/*HER2* ^amp^ (3%) and TNBC (3%) cohorts. *AR* was amplified in 1% in all three groups of MBCs. AR protein expression was not assessed in this study.

**FIGURE 2:**
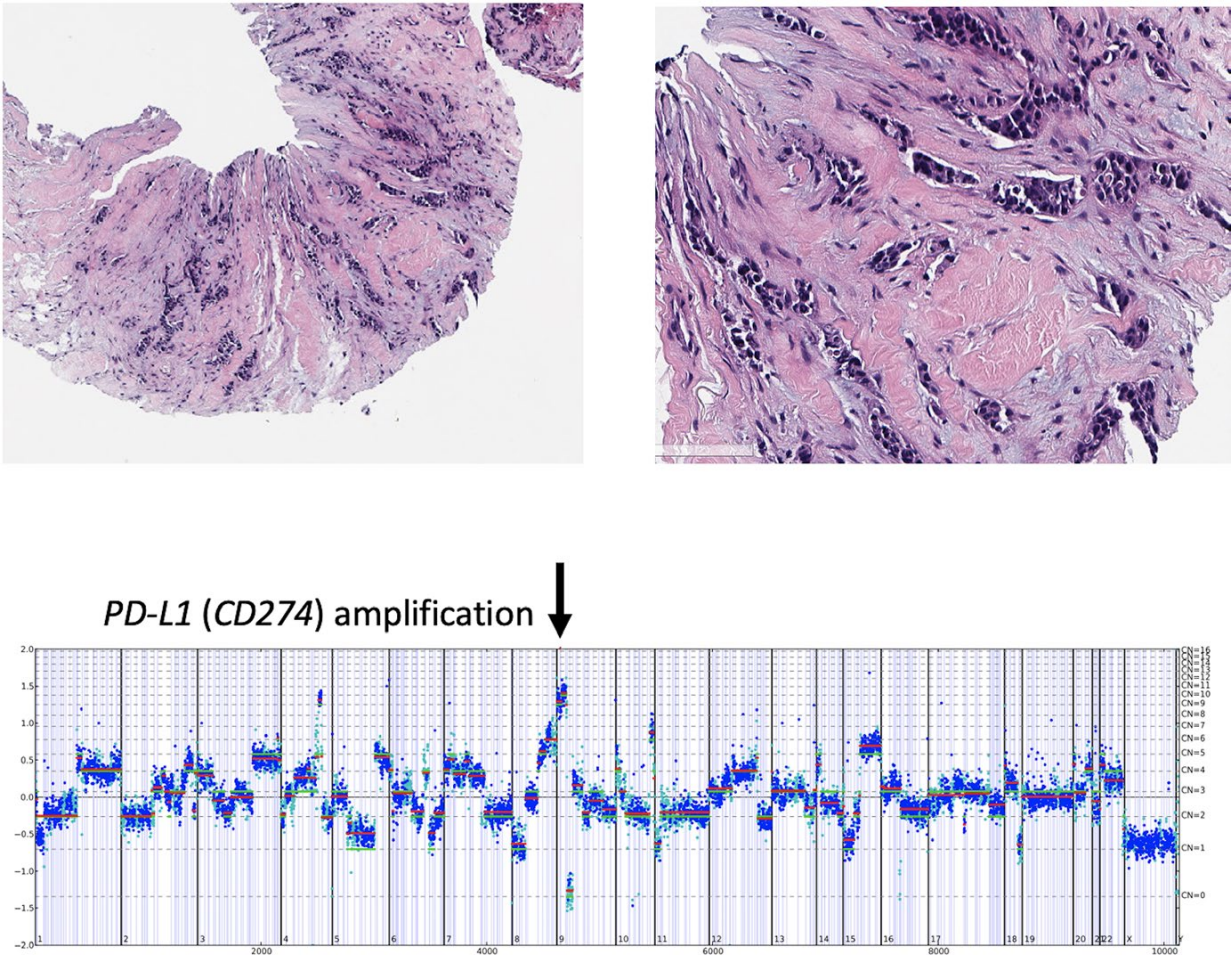
Case of stage IV triple‐negative breast cancer in a 72‐year‐old woman with a *CD274* amplification. The genome‐wide copy number plot demonstrates an amplification of the *CD274* gene at 12 copies per cell

**FIGURE 3:**
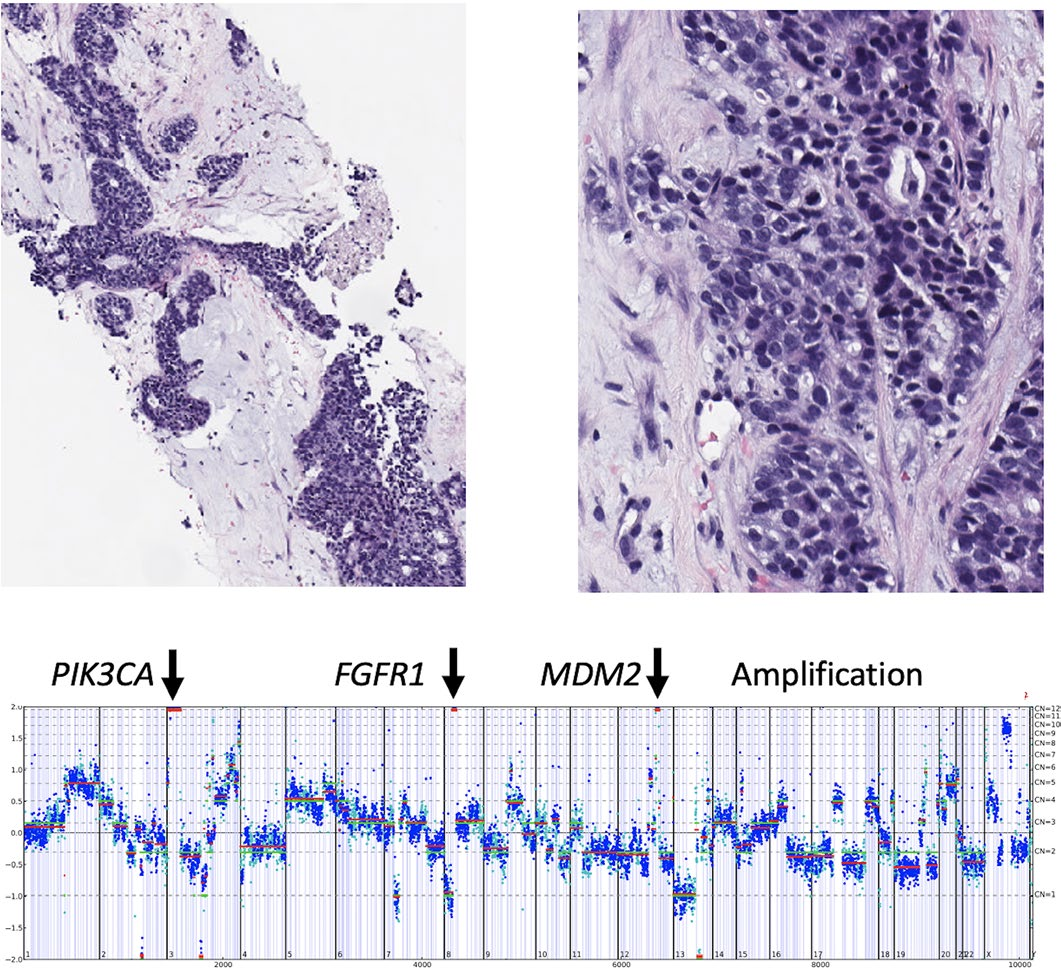
Case of stage IV triple‐negative breast cancer in a 57‐year‐old patient with *MDM2* amplification. Comprehensive genomic profiling revealed multiple potentially targetable gene amplifications in *FGFR1* and *PIK3CA*, but also featured amplification of *MDM2*

When focused on potential biomarkers of ICPI responsiveness, this study includes individual GA, calculated MSI status, TMB derived from CGP, and PD‐L1 expression determined by IHC. Individual genomic markers that have been linked to responsiveness to ICPI in other tumor types were identified infrequently in MBC. This included markers of potential ICPI benefit such as *CD274* (PD‐L1) amplification found in 1%–3% of the MBC (Figure 2). *BRAF*‐activating SV mutations are found in 1%–4% of MBC, and *PBRM1*‐inactivating SV mutations are found in 1% of MBC. MSI‐H status, predictive of efficacy for the ICPI pembrolizumab for all solid tumors including MBC, was uncommon in this study, ranging from 0.1% to 0.4%. The calculated TMB, also linked to responsiveness of advanced malignancies to ICPI in multiple studies, varied among the three MBC groups. When ≥10 mutations/Mb is used as a cut‐off, 8%–12% of MBC were positive. Using the VENTANA PD‐L1 SP142 Assay that was included in the approval of the atezolizumab‐nab‐paclitaxel combination treatment for TNBC in March of 2019, the frequency of positive staining ranged from 13% in the ER^pos^/*HER2* ^neg^ subgroup to 33% in the ER^neg^/*HER2* ^amp^ subgroup to 47% in the TNBC subgroup. This study also evaluated biomarkers associated with resistance and hyperprogression to ICPI and included inactivating GA in *STK11* found in 1%–2% of MBC and amplification of the *MDM2* gene encountered in 3%–6% of MBC (Figure 3).

## 4 DISCUSSION

Currently, the US FDA‐approved companion diagnostic biomarkers linked to ICPI for breast cancer include PD‐L1 expression using the VENTANA PD‐L1 (SP142) IHC assay in TNBC for atezolizumab, TMB detected by CGP using the FoundationOne CDx assay for pembrolizumab, in addition to the identification of MSI/mismatch repair deficiency (dMMR) by multiple modalities for pembrolizumab. In this study, the metastatic tumor tissues of a significant percentage of patients with MBC were found to contain these GAs associated with response to ICPIs. Among patients in the ER^pos^/*HER2* ^neg^ subgroup, 46.2% were found to harbor US FDA‐approved targetable alterations, including *PIK3CA* (38%), TMB ≥10 mut/Mb (8%) and MSI‐H (0.2%). Within the ER‐/HER2‐amplified cohort, 12.1% possessed approved targetable alterations, including TMB ≥10 mut/Mb (12%) and MSI‐H (0.1%). The highest percentage of actionable mutations was found in the historically difficult to treat TNBC group, with 56.4% of patients possessing GAs indicative of response to ICPIs including PD‐L1 SP142 staining ≥1% (47%), TMB ≥10 mut/Mb (9%), and MSI‐H (0.4%). *PIK3CA* frequency was 19% in the TNBC group.

The majority of studies involving breast cancer and ICPI have focused on PD‐1 and PD‐L1 blockades.^21^ The KEYNOTE‐012 trial (phase 1b) studied pembrolizumab in advanced, heavily pretreated TNBC. In the 111 enrolled TNBC patients, 58.6% of their tumors had some level of PD‐L1 expression. Among 27 patients for whom response was analyzed, the ORR was 18.5% and the irAE was reportedly mild.^27^ The phase Ib KEYNOTE‐028 trial showed that in PD‐L1+ ER+/HER2‐negative MBC, pembrolizumab had an ORR of 12%.^21^ Another phase 1b/II trial showed that combination of pembrolizumab and eribulin had a favorable response, but the PD‐L1 status did not predict the response to treatment.^16^ I‐SPY2 (Investigation of Serial Studies to Predict Your Therapeutic Response with Imaging and molecular Analysis 2) and phase III KEYNOTE‐522 are some of the studies evaluating ICPIs, such as pembrolizumab, in combination with standard chemotherapy.^21^ The results of the latter showed that the combination of pembrolizumab and chemotherapy led to a pathological complete response frequency of 64.8% when compared to 51.2% with placebo using chemotherapy.^28^ It has been observed that HER2 expression can result in immune suppression, so trials like PANACEA and PembroMab are evaluating pembrolizumab, trastuzumab, and ado‐trastuzumab emtansine in HER2+ disease.^21^, ^29^ KEYNOTE‐119 is an ongoing phase 3 trial comparing monotherapy with pembrolizumab and chemotherapy in metastatic TNBC.^30^ Pembrolizumab monotherapy was reported to not significantly improve OS as second‐ or third‐line treatment for mTNBC versus chemotherapy, although the pembrolizumab treatment effect increased as PD‐L1 enrichment increased.^31^ Several trials have also been performed with the monoclonal antibody atezolizumab which prevents the interaction of PD‐L1 with PD‐1 and B7.1 (CD80), which in turn causes less suppression of tumor‐reactive lymphocytes.^32^

PD‐L1 expression itself is seen as a promising biomarker for breast cancer in general. Its expression has been shown to be associated with lymph node spread, higher histological grades, ER receptor negativity, and TNBC.^33^ In the IMpassion130 study by Schmidt et al, PD‐L1 expression was around 41%.^34^ In most studies of TNBC IHC staining of ICs, PD‐L1 expression has ranged from 40% to 65%.^35^ In 2014, Schalper et al reported that nearly 60% of breast cancers expressed PD‐L1 in ICs and that the IC PD‐L1 expression status was associated with better overall disease outcomes.^36^ In the current study, PD‐L1 expression was 47% using the VENTANA PD‐L1 SP142 Assay. In both the current study and the study reported by Schmid et al,^34^ a cut‐off of >1% expression in ICs was used to define positive PD‐L1 expression. Currently, there is no standard method of staining or cut‐off of positive PD‐L1 expression in ER+and HER2‐amplified MBC including whether to include the IHC expression in IC only, TC only, or the combination.^34^, ^35^ Since VENTANA PD‐L1 SP142 was approved as a CDx for treatment of TNBC with atezolizumab and nab‐paclitaxel, we decided to use the same assay for all MBC samples; hence, we are only scoring IC staining.

PD‐L1, the protein encoded by the *CD274* gene, undergoes several genetic alterations including amplification, epigenetic regulation, transcriptional activation, glycosylation, phosphorylation, and ubiquitination. Copy number alterations (CNAs) resulting in amplification of *CD274* in Hodgkin's lymphoma are associated with high response rates to PD‐L1 inhibitors.^36^ Barrett et al evaluated 54 cases of TNBC and found that high levels of PD‐1 and PD‐L1 with high copy number variants (CNVs) were found in 20% and 37%, respectively. This rate contrasts with the current study where *CD274* amplification was identified in only 1%–3% of MBC cases. A large analysis of 118 187 tumor samples showed that *CD274* amplification was identified in 0.7% of the samples and did not correlate with PD‐L1 expression.^16^

In this study, 5%–15% of MBC cases possessed *PTEN* alterations. Prior work has shown the loss of the tumor suppressor *PTEN* to be associated with poor responses to PD‐1 blockade in patients with melanoma, uterine sarcoma, and mTNBC,^19^ and that partial *PTEN* deletions associate with worse OS in breast cancer.^37^ In addition to the established role of PTEN in cancer progression, PTEN deficiency can lead to increased immunosuppressive cytokines and expression signatures that are unfavorable for effective antitumor responsiveness.^38^, ^39^, ^40^ In breast cancer cell lines, Mittendorf et al showed that *PTEN* loss resulted in overexpression of PD‐1 and PD‐L1 which was downregulated after PI3K pathway inhibition and may increase antitumor adaptive processes.^41^ Clinical trials are needed to confirm that PTEN‐altered breast cancer harbors ICPI resistance that may be reversed by PI3K/AKT/mTOR inhibitor mono‐ or combination therapy.

*PTEN* deletion and high TMB were not associated with PD‐L1 expression in the current analysis.^42^ The JAK2 pathway also seems to play a role in regulating PD‐L1 expression and reports are emerging showing that JAK2 inhibitors may have a benefit in TNBCs with 9p.24.1 amplification.^43^ Even though numerous studies have included the status of *CD274* amplification, data on its impact on clinical outcomes and ICPI therapy are very limited, highlighting the need for more research on this topic in the future.

Findings from the various KEYNOTE trials in other tumor types led to the accelerated approval by the FDA of pembrolizumab in MSI‐H solid tumors. A large study of 39 cancer types has found an MSI‐H status in 1.53% of primary breast cancers, in comparison to our study where it was only 0.1%–0.4% in cases of clinically advanced and metastatic disease.^44^ Given the low prevalence of MSI in breast cancer and lack of robust data on clinical outcomes, MSI, at least for now, seems to have limited utility in breast cancer.

Tumors with higher TMB have shown enhanced responses to ICPIs. In the recent CheckMate 026 trial, NSCLC patients with high TMB had a better ORR and PFS on treatment with nivolumab than with chemotherapy.^45^ Thomas et al. found that immune subtypes with high TMB had better ORR to ICPI therapies than subtypes with low TMB levels in *BRCA*‐positive breast cancer. In this study, the TMB was measured by whole genome sequencing (WGS) and ranged from 0 to 115 mutations/Mb with a mean of 1.63 mutations/Mb, which was used as the cut‐off.^46^ In WGS analysis by Park et al, 100 mutations/Mb was considered as high TMB.^47^ TMB varies widely among other cancers also reflecting the technique used for measurement. For example, the CheckMate 026 trial in lung cancer used whole exome sequencing and a TMB score >243 mutations/Mb as the cut‐off.^45^ In breast cancer, some reports suggest that TMB is higher in ER‐negative cancers when compared to ER‐positive cancers.^48^ High TMB has also been associated with poor disease‐free survival.^49^ More work is needed to understand the relationship of clinical response along the continuum of TMB scores and how discrete values can provide more clinical information beyond a simple dichotomous classification. Additionally, harmonization efforts are currently underway to ensure alignment and improve interchangeability between TMB estimates generated from different targeted gene panels,^47^ an activity essential to ensure this biomarker provides consistent information to further evaluate its clinical implications in controlled prospective studies and to inform treatment decisions across diagnostic platforms.

*BRAF* mutations are very uncommon in breast cancer representing around 0%–1.2% in public databases of both localized curable primary and MBC and 1%–4% of clinically advanced MBC in the current study. Reports indicate that TMB in *BRAF*‐altered MBC is generally higher, and hence may be a potential biomarker of ICPI efficacy, but larger studies are lacking with regard to the predictive status of *BRAF* mutations in breast cancer. Inactivation of *STK11* has been shown to cause a “cold” tumor immune microenvironment by blunting the action of cytotoxic T lymphocytes and has been associated with PD‐1/PD‐L1 inhibitor resistance in NSCLC.^18^ Traditionally, *STK11* alterations in breast cancer, observed at 1%–2% in this study, have been associated with Peutz‐Jeghers syndrome and have not, to date, been linked to resistance to ICPI treatments.

Finally, a single study analyzed the molecular profiles of 102 878 diverse cancer types and found that *MDM2* amplification occurred in 3.5% of the patients.^46^ Hyperprogression occurs in 10–30% of cancers treated with immunotherapy and has been associated with *MDM2* amplification. Reports have shown significant correlation with failure of anti‐PD‐1/PD‐L1 agents and *MDM2* amplification, but few of these have been specific to breast cancer.^46^, ^50^ More studies that analyze response to ICPIs in MBC of various types are needed to confirm if *MDM2* amplification is a predictor of resistance and hyperprogression in MBC patients treated with ICPI agents.

Both our results and published literature suggest that in addition to guiding targeted therapy selection, CGP shows potential to identify GA linked to response and resistance to ICPI therapy in MBC. The demonstration of clinical benefit of immune checkpoint blockade in MBC supports the need for the development of biomarkers used to guide the use of ICPI drugs for these patients.

## CONFLICT OF INTEREST

Abirami Sivapiragasam: No conflict to disclose. P. Ashok Kumar: No conflict to disclose. Ethan S. Sokol: Employment by Foundation Medicine Inc. Stock in F. Hoffman La Roche Ltd. Lee A. Albacker: Employment by Foundation Medicine Inc. Stock in F. Hoffman La Roche Ltd, J. Keith Killian: Employment by Foundation Medicine Inc. Stock in F. Hoffman La Roche Ltd. Shakti Ramkissoon: Employment by Foundation Medicine Inc. Stock in F. Hoffman La Roche Ltd. Richard Huang: Employment by Foundation Medicine Inc. Stock in F. Hoffman La Roche Ltd. Patents with VENTANA (Roche). Eric A. Severson: Employment by Foundation Medicine Inc. Stock in F. Hoffman La Roche Ltd. Natalie Danziger: Employment by Foundation Medicine Inc. Charlotte A. Brown: Employment by Foundation Medicine Inc. Stock in F. Hoffman La Roche Ltd. Kimberly McGregor: Employment by Foundation Medicine Inc. Stock in F. Hoffman La Roche Ltd. Jeffrey S. Ross: Employment by Foundation Medicine Inc. Stock in F. Hoffman La Roche Ltd.

## Supporting information

Table S1

Table S2

Table S3

Table S4

## Data Availability

The data that support the findings of this study are available in the supplementary material of this article.
