# Supplementary material for: Predictive Biomarkers for Immune Checkpoint Inhibitors in Metastatic Breast Cancer
Source: Cancer Med. 2020 Dec 12;10(1):53–61. doi: 10.1002/cam4.3550 (PMC7826457; doi:10.1002/cam4.3550)
Supplement: Supplementary file 1 — Table S1 [file CAM4-10-53-s001.docx]

Supplementary Table 1 – Sites of metastasis in the MBC cohort

|  | **ER^pos^/HER2^neg^** | | **ER^neg^/HER2^amp^** | | **TNBC** | | **ER^pos^/HER2^neg^ vs. ER^neg^/HER2^amp^** | **ER^pos^/HER2^neg^ vs. TNBC** | **ER^neg^/HER2^amp^ vs. TNBC** |
| --- | --- | --- | --- | --- | --- | --- | --- | --- | --- |
| **Metastatic Sample Site** | **# cases** | **%** | **# cases** | **%** | **# cases** | % | **p value** | **p value** | **p value** |
| Abdominal wall | 2 | 0.2886 | 2 | 0.1633 | 3 | 1.2245 | 0.6232 | 0.1151 | 0.0352 |
| Adrenal gland | 1 | 0.1443 | 4 | 0.3265 | 2 | 0.8163 | 0.6594 | 0.1687 | 0.2631 |
| Bone | 75 | 10.8225 | 57 | 4.6531 | 8 | 3.2653 | **<0.0001** | **0.0002** | 0.3974 |
| Brain | 11 | 1.5873 | 109 | 8.8980 | 14 | 5.7143 | **<0.0001** | **0.0017** | 0.1282 |
| Cervix and Uterus | 2 | 0.2886 | 5 | 0.4082 | 1 | 0.4082 | 1.0000 | 1.0000 | 1.0000 |
| Chest Wall | 39 | 5.6277 | 75 | 6.1224 | 17 | 6.9388 | 0.6888 | 0.4371 | 0.6643 |
| Stomach, Small Intestine and Colon | 4 | 0.5772 | 10 | 0.8163 | 1 | 0.4082 | 0.7812 | 1.0000 | 0.7027 |
| Head and Neck | 7 | 1.0101 | 25 | 2.0408 | 1 | 0.4082 | 0.0977 | 0.6881 | 0.1066 |
| Liver | 270 | 38.9610 | 315 | 25.7143 | 48 | 19.5918 | **<0.0001** | **<0.0001** | **0.0427** |
| Lung | 29 | 4.1847 | 139 | 11.3469 | 18 | 7.3469 | **<0.0001** | 0.0606 | 0.0697 |
| Lymph Node | 104 | 15.0072 | 243 | 19.8367 | 69 | 28.1633 | **0.0094** | **0.0000** | **0.0047** |
| Mediastinum | 3 | 0.4329 | 4 | 0.3265 | 2 | 0.8163 | 0.7083 | 0.6101 | 0.2631 |
| Soft tissue | 56 | 8.0808 | 74 | 6.0408 | 23 | 9.3878 | 0.0896 | 0.5065 | 0.0658 |
| Ovary | 3 | 0.4329 | 2 | 0.1633 | 1 | 0.4082 | 0.3587 | 1.0000 | 0.4215 |
| Pancreas | 0 | 0.0000 | 2 | 0.1633 | 0 | 0.0000 | 0.5382 | 1.0000 | 1.0000 |
| Pelvis | 3 | 0.4329 | 3 | 0.2449 | 0 | 0.0000 | 0.6737 | 0.5715 | 1.0000 |
| Peritoneum, Retroperitoneum and Omentum | 5 | 0.7215 | 6 | 0.4898 | 2 | 0.8163 | 0.5397 | 1.0000 | 0.6272 |
| Pericardium, Pleura and Pleural Fluid | 27 | 3.8961 | 30 | 2.4490 | 9 | 3.6735 | 0.0921 | 1.0000 | 0.2766 |
| Skin | 47 | 6.7821 | 109 | 8.8980 | 24 | 9.7959 | 0.1174 | 0.1590 | 0.6270 |
| Thyroid | 2 | 0.2886 | 0 | 0.0000 | 0 | 0.0000 | 0.1304 | 1.0000 | 1.0000 |
| Trachea | 1 | 0.1443 | 0 | 0.0000 | 0 | 0.0000 | 0.3613 | 1.0000 | 1.0000 |
| Unknown | 2 | 0.2886 | 11 | 0.8980 | 2 | 0.8163 | 0.1522 | 0.2805 | 1.0000 |
